# Point-of-care testing for coeliac disease: primary care diagnostic technology update

**DOI:** 10.3399/bjgp13X668401

**Published:** 2013-05-28

**Authors:** Jaspreet Khangura, Ann Van den Bruel, Rafael Perera, Carl Heneghan, Christopher P Price, Jane Wolstenholme, Matthew Thompson, Annette Plüddemann

**Affiliations:** Horizon Scanning Programme, Centre for Monitoring and Diagnosis, University of Oxford, Oxford.; Horizon Scanning Programme, Centre for Monitoring and Diagnosis, University of Oxford, Oxford.; Horizon Scanning Programme, Centre for Monitoring and Diagnosis, University of Oxford, Oxford.; Horizon Scanning Programme, Centre for Monitoring and Diagnosis, University of Oxford, Oxford.; Horizon Scanning Programme, Centre for Monitoring and Diagnosis, University of Oxford, Oxford.; Horizon Scanning Programme, Centre for Monitoring and Diagnosis, University of Oxford, Oxford.; Horizon Scanning Programme, Centre for Monitoring and Diagnosis, University of Oxford, Oxford.; Horizon Scanning Programme, Centre for Monitoring and Diagnosis, University of Oxford, Oxford.

**Clinical Question**

In primary care patients with suspected coeliac disease, what is the accuracy and utility of point-of-care (POC) testing for coeliac disease compared to standard practice?

## BACKGROUND AND ADVANTAGES OVER EXISTING TECHNOLOGY

Coeliac disease is a malabsorption syndrome precipitated by gluten ingestion, and characterised by inflammation of the small intestine.

Serological tests for IgA anti-tissue transglutaminase antibody (tTGA) and anti-endomysial antibody (EMA) have high sensitivity and specificity for coeliac disease.^1^,^2^ In patients with IgA deficiency, the IgG class of the tTGA and EMA tests are recommended. Anti-gliadin antibodies are no longer used for the detection of coeliac disease, except in children younger than 18 months.^2^ Conventional serological tests are performed in central laboratories, whereas the point-of-care (POC) test can be performed in practice or at home. Both the POC and conventional serological tests require patients to be on a normal gluten-containing diet at the time of testing, since IgA-tTGA titres diminish on a gluten-free diet.

## DETAILS OF TECHNOLOGY

Two POC devices available on the market were identified.

### Biocard Coeliac Test Kit (Ani Biotech, Finland; UK Distributor: BHR Pharmaceuticals Ltd)

There are two versions of the test, a home test and a professional test; a ‘total IgA measuring system’ is included in the professional kit. The Biocard requires a drop of whole blood (finger-prick) and provides results within 10 minutes.

Anti-tTG IgA antibodies bind to antigen in the test strip to form a visible line. A positive test result shows two lines, while only one line appears if the test is negative. If there is no line, IgA deficiency should be suspected.

### Stick CD1 and CD2 (Operon SA, Zaragoza, Spain)

Both are one-step tests detecting IgA, IgG, and IgM antibodies against human tTG; and the CD2 test also detects anti-gliadin antibodies. The tests use serum instead of whole blood, which limits their applicability to ambulatory settings. Results are available within 10 minutes.

## PATIENT GROUP AND USE

- Patients in whom coeliac disease is suspected because of signs or symptoms (chronic or intermittent diarrhoea, failure to thrive [children], persistent or unexplained gastrointestinal (GI) symptoms such as nausea and vomiting, prolonged fatigue, recurrent abdominal pain, cramping or distension, sudden/unexpected weight loss, unexplained iron-deficiency anaemia).^2^
- Patients with coexisting conditions associated with coeliac disease, such as autoimmune thyroid disease, dermatitis herpetiformis, irritable bowel syndrome, type 1 diabetes, or who have first-degree relatives with coeliac disease.^2^
- The Biocard test is not suitable for children <5 years of age or for patients with IgA deficiency.

## IMPORTANCE

The prevalence of coeliac disease in the UK is estimated to be 0.8%–1.9% in the general population, and 4.5%–12% among first-degree relatives.^2^ Studies show an average of more than 10 years from symptom onset to diagnosis.^3^ Undiagnosed coeliac disease can lead to chronic illness including anaemia and osteoporosis (with resulting increased risk of fractures).^2^ In children, undiagnosed coeliac disease can result in growth failure, delayed puberty, and dental problems.

## PREVIOUS RESEARCH

### Accuracy compared to existing technology

Four case-control studies used the Biocard test in a population of biopsy-confirmed patients with coeliac disease and laboratory controls.^4^–^7^

The earliest study was based on 121 consecutive samples from patients and 107 controls who had normal villous morphology on pathology.^5^ Biocard and laboratory serum tests (EMA and tTGA) were compared to the gold standard of duodenal biopsy. The Biocard test gave a sensitivity of 97% and a specificity of 94%, (positive likelihood ratio [LR] 14.9 and negative LR 0.35). By comparison, laboratory serum EMA and tTGA tests had a sensitivity of 97% and 99%, respectively, and both showed a specificity of 100%.

Subsequently, the Biocard test was evaluated in 24 untreated patients with coeliac disease and 19 controls compared to duodenal biopsy, with a sensitivity of 92% and specificity of 79%.^6^ A third study^7^ investigated the Biocard test in 139 consecutive untreated patients and 103 controls. Sensitivity and specificity were 93% and 94%, respectively, compared with duodenal biopsy. None of the patients in the above studies were IgA deficient. The fourth and final case-control study showed 90% sensitivity and 100% specificity compared with serological laboratory testing.^4^

The diagnostic accuracy of the Biocard test was also assessed in a cross-sectional study of 150 patients from a tertiary clinic with suspected but unconfirmed coeliac disease.^5^ Compared with serological EMA and tTGA tests the Biocard results were found to be concordant in 145 of 150 patients. The sensitivity and specificity of the Biocard test relative to both EMA and tTGA serological tests were 97% and 96%, respectively.

One study assessed the Biocard test for screening.^8^ District nurses at primary care centres in Hungary tested 6-year-old children (*n* = 2676) and offered biopsy if any result was positive. Coeliac disease was confirmed in 32 children (1.2%). When compared to biopsy plus follow-up, Biocard test sensitivity was 78% and specificity was 99.8%.

One study^4^ compared the Stick CD1 test and the Biocard test. The sensitivity of the Stick CD1 test was 100% (including 4 IgA deficient coeliac disease patients) with 95% specificity while the Biocard sensitivity was 90% with 100% specificity.

One prospective multicentre study evaluated the accuracy of Stick CD1 and CD2 test in 113 children with confirmed coeliac disease.^9^ For the CD1 Stick test (tTGAs), sensitivity was 97% and specificity was 99%. CD2 displayed a sensitivity of 95% and a specificity of 99% for tTGAs and a sensitivity of 63% and a specificity of 95% for gliadin antibodies.

### Impact compared to existing technology

The diagnostic accuracy of the Biocard test is high (sensitivity between 90–97% and specificity between 79–100%), but this may be an overestimate due to the study designs and the selective populations. Sensitivity in asymptomatic children was much lower (65–79%), which means that the ability to rule out coeliac disease in these children is reduced. There is limited evidence available for the Stick coeliac disease tests.

Patient self-testing raises other concerns: those who self-diagnose may begin gluten-free diets without confirmatory testing, appropriate nutritional advice, or medical investigation for complications or comorbid conditions associated with coeliac disease.

Given that 8% of patients with coeliac disease are IgA deficient, the Biocard test may give false negative results in this group, leading to delays in seeking medical attention.

Use of the Biocard test by professionals may be of value in situations where venepuncture is difficult, for example, in children.

### Cost-effectiveness and economic impact

Early-case identification through POC testing has the potential to prevent gluten-related morbidity and reduce costs. However, one UK study^10^ found increased healthcare costs before and after diagnosis in patients with coeliac disease compared with controls, while two studies from the US^11^,^12^ reported that an increased rate of coeliac disease diagnosis led to reduced healthcare service utilisation and costs. None of these studies included quality of life measures, and there is no evidence on the cost-effectiveness of using POC testing to screen for coeliac disease in primary care.

#### Relevant Guidelines

The 2009 NICE guideline CG 86 recommends that self-tests and/or POC tests for coeliac disease should not be used as a substitute for laboratory-based tests, and that patients with positive self- or POC-tests are sent for further serological testing.^2^ NICE advises, based on an evaluation of one early POC test, that ‘limited evidence suggests that point-of-care tests and self tests may be accurate but require further evaluation’.^2^

#### What this technology adds

Although the evidence is limited, point-of-care testing may be helpful in the diagnostic work-up of coeliac disease in primary care settings by increasing speed of results or access to testing in some settings, as sensitivity and specificity are comparable with laboratory-based serology. However, a negative result does not safely rule out coeliac disease.

#### Methodology

Standardised methodology was applied in writing this report, using prioritisation criteria and a comprehensive, standardised search strategy, and critical appraisal. Full details of these are available from www.madox.org.
